# Zinc treatment ameliorates diarrhea and intestinal inflammation in undernourished rats

**DOI:** 10.1186/1471-230X-14-136

**Published:** 2014-08-05

**Authors:** Camila AA de Queiroz, Said Gonçalves C Fonseca, Priscila B Frota, Ítalo L Figueiredo, Karoline S Aragão, Carlos Emanuel C Magalhães, Cibele BM de Carvalho, Aldo Ângelo M Lima, Ronaldo A Ribeiro, Richard L Guerrant, Sean R Moore, Reinaldo B Oriá

**Affiliations:** 1 Laboratory of the Biology of Tissue Healing, Ontogeny and Nutrition, Department of Morphology and Institute of Biomedicine, School of Medicine, Federal University of Ceara, Rua Cel. Nunes de Melo, 1315, Fortaleza, CE, Brazil; 2 Department of Physiology and Pharmacology and Institute of Biomedicine, School of Medicine, Federal University of Ceara, Rua Cel. Nunes de Melo, 1315, Fortaleza, CE, Brazil; 3 Department of Microbiology and Institute of Biomedicine, School of Medicine, Federal University of Ceara, Rua Cel. Nunes de Melo, 1315, Fortaleza, CE, Brazil; 4 Department of Pharmacy, Faculty of Pharmacy, Odontology, and Nursing, Federal University of Ceara, Rua Capitão Francisco Pedro, 1210, Fortaleza, CE, Brazil; 5 Center for Global Health, Division of Infectious Diseases and International Health, University of Virginia, Charlottesville, VA, USA; 6 Division of Gastroenterology, Hepatology, & Nutrition, Cincinnati Children’s Hospital Medical Center, Cincinnati, OH, USA

**Keywords:** Zinc, Malnutrition, Diarrhea, Inflammation, Bacterial translocation

## Abstract

**Background:**

WHO guidelines recommend zinc supplementation as a key adjunct therapy for childhood diarrhea in developing countries, however zinc’s anti-diarrheal effects remain only partially understood. Recently, it has been recognized that low-grade inflammation may influence stunting. In this study, we examined whether oral zinc supplementation could improve weight, intestinal inflammation, and diarrhea in undernourished weanling rats.

**Methods:**

Rats were undernourished using a northeastern Brazil regional diet (RBD) for two weeks, followed by oral gavage with a saturated lactose solution (30 g/kg) in the last 7 days to induce osmotic diarrhea. Animals were checked for diarrhea daily after lactose intake. Blood was drawn in order to measure serum zinc levels by atomic absorption spectroscopy. Rats were euthanized to harvest jejunal tissue for histology and cytokine profiles by ELISA. In a subset of animals, spleen samples were harvested under aseptic conditions to quantify bacterial translocation.

**Results:**

Oral zinc supplementation increased serum zinc levels following lactose-induced osmotic diarrhea. In undernourished rats, zinc improved weight gain following osmotic diarrhea and significantly reduced diarrheal scores by the third day of lactose intake (p < 0.05), with improved jejunum histology (p < 0.0001). Zinc supplementation diminished bacterial translocation only in lactose-challenged undernourished rats (p = 0.03) compared with the untreated challenged controls and reduced intestinal IL-1β and TNF-α cytokines to control levels.

**Conclusion:**

Altogether our findings provide novel mechanisms of zinc action in the setting of diarrhea and undernutrition and support the use of zinc to prevent the vicious cycle of malnutrition and diarrhea.

## Background

The vicious cycle of malnutrition and enteric infections in poor settings of the developing world may cause an environmental enteropathy postulated to result from the combined effects of marginal diets, unsanitary environments, and repeated and persistent enteric infections [1,2]. Recent data from studies in developing countries, including Brazil and Peru, have documented a lasting impact of diarrhea (as with malnutrition and intestinal helminthic infections) on child development with ill effects on cognition, growth, and educational performance [3-5].

In addition, the vicious cycle of childhood undernutrition and infection may impair the efficacy of oral vaccines against life-threatening enteric pathogens and therefore amplify this loop of intestinal barrier breakdown, bacterial translocation, and inflammation leading to poor nutrient absorption [6,7]. All of this could potentially increase the global DALY (disability adjusted life years) due to diarrheal diseases or enteric infections to a level not previously considered [8]. Any improvements in water sanitation, food security, and antimicrobials may lead to better mucosal immunity, adapted gut microbiome, and improved intestinal barrier function. All these factors are key to reduce the long-term and devastating effects of this vicious cycle on children’s development, therefore helping to achieve full human potential.

Zinc is an essential component of many enzymes and is necessary for the activity of many others [9]. Zinc also has a role in the acute phase of inflammation and the immune response, although the mechanisms through which it acts are still unclear [10]. Zinc participates in DNA and RNA syntheses, which directly correlate with cellular replication, chondrocyte and osteocyte differentiation, and cellular transcription. In addition, it is a key co-factor for IGF-1, collagen, osteocalcin, and alkaline phosphatase syntheses [11].

Zinc supplementation lowers the risk of diarrhea in children [12]. Additionally, chronic diarrhea causes zinc deficiency, which further contributes to diarrhea [13,14]. The Duration and severity of diarrheal diseases and immunosuppression in undernourished children from developing countries are greater than their nourished counterparts. All these factors can be associated with zinc deficiency because zinc supplementation improves theses outcomes. [15].

Recently, it has been recognized that a chronic low-grade systemic pro-inflammatory state may influence stunting in children [16], which may be caused by intestinal barrier disruption and bacterial translocation [17]. Zinc supplementation has been shown beneficial to improve chronic inflammation [18,19] and may have a role in protecting undernourished children with acute diarrhea. In this study, we have addressed whether zinc supplementation could benefit weight gain, intestinal inflammation and gut-to-blood bacterial translocation in weanling rats challenged by undernutrition, with or without lactose-induced osmotic diarrhea.

## Methods

### Animals

We used 60 weaned male Wistar rats, weighing 40-60 g, provided by the Department of Physiology and Pharmacology/Federal University of Ceará. Immediately after weaning (21 days old), experimental rats were randomly divided in five groups, as following: nourished controls, undernourished controls, undernourished controls challenged with saturated lactose, undernourished rats receiving zinc supplementation, and undernourished and lactose challenged rats receiving zinc supplementation. All animals had free access to food and water and were monitored daily for weight gain.

Study protocols were in accordance with the Brazilian College for Animal Care guidelines and were approved by the Federal University of Ceará Animal Care and Use Committee.

### Experimental undernutrition

During 14 days post-weaning, nourished control rats received standard chow diet while undernourished control rats received isocaloric Brazilian northeastern regional basic diet (RBD). In the last 7 days, these two groups received 2 ml of PBS by gavage. The RBD is a well-studied rodent diet high in carbohydrates and marginally deficient in protein, fat, and minerals. It was formulated according to Teodosio et al. [20] to represent the multideficient diet of poor populations in northeastern Brazil. RBD and commercial chow (Purina®) diets present the following nutrients, respectively: protein (9.35% and 20.30%), carbohydrate (70.60% and 56.0%), and fat (0.36% and 3.33%). The RBD has been shown to reduce growth velocity and to cause intestinal barrier disruption in suckling mice [21].

### Induction of osmotic diarrhea and zinc treatment

In order to evaluate the effect of zinc supplementation on acute diarrhea, lactose-driven osmotic diarrhea was induced to the study rats, as described by Teichberg et al. [22]. A subset of undernourished rats that received RBD was also challenged by a saturated lactose solution (monohydratated sodium lactose, Vetec Química, Rio de Janeiro, Brazil), which was given by gavage diluted in 2 mL of PBS, 30 g/Kg, starting on day 8 until day 14 to induce osmotic diarrhea. Non-lactose challenged groups received PBS at the same volume by gavage in the last 7 days of the experiment as well. Zinc-treated rats received zinc acetate (Sigma, São Paulo, Brazil) in the drinking water (500 mg/L) on day 8 until day 14 in order to determine whether zinc could reduce diarrheal episodes. Zinc supplementation solution was chosen based on a previous study in mice showing improvements in the immune system [23] and after a pilot done to weanling Wistar rats confirming improvements in weight gain and zinc serum levels (data not shown).

All experimental rats were euthanized by cervical dislocation on day 15 of the experiment, after being previously anesthetized with ketamine (8 mg/100 g) and xylazin (0.8 mg/100 g).

### Diarrhea scores

**Figure 1:**
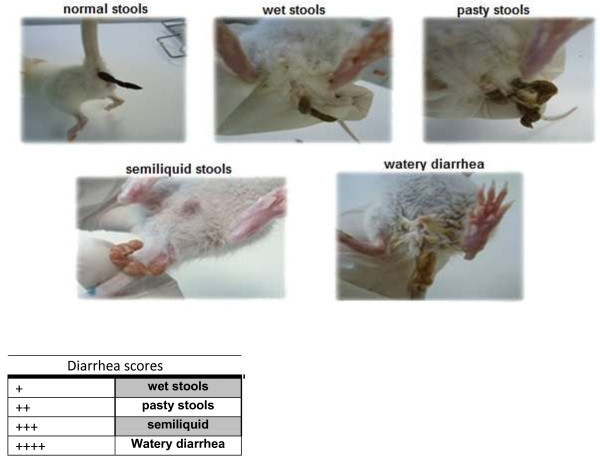
**Diarrheal scores from experimental rats.** Scores were obtained by checking for diarrhea daily by a trained veterinarian, 24 h after lactose intake by gavage, at the same time of the day for all groups**.** Scoring criteria accounted for changes in stool consistency and humidity, as follows: 0- normal stool consistency and dryness; 1-wet stools; 2-pasty stools; 3-semiliquid stools; 4-watery diarrhea.

To quantify the incidence of daily diarrhea, a single examination was conducted 24 hours after the lactose gavage (30 g/kg/day) by a trained veterinarian who performed a clinical evaluation of the lactose-challenged rats and confirmed and scored the osmotic diarrhea episode. Diarrheal scores were defined according to stool consistency, appearance, and humidity. Animals with watery diarrhea were considered with the maximum score (++++). The determination of diarrhea scores was performed according to the Figure 1.

### Serum zinc measurement

In order to evaluate whether RBD with or without lactose-induced diarrhea could cause zinc deficiency, we measured serum zinc levels in the experimental rats. Blood was collected by intracardiac puncture. Analyses of serum zinc levels were conducted in the Clementino Fraga Laboratory, using atomic absorption spectroscopy (SpectrAA 55 AAS, Varian, CA, USA). A standard calibration curve was obtained using a standard zinc solution (0.2 mg Zn/L; J.T. Baker), as described elsewhere [24].

### ELISA cytokine assay

Since inflammation could cause intestinal barrier impairment, we also assessed key intestinal cytokines previously shown to be affected by zinc supplementation [18]. Specimens were stored at −80°C until required for assay. The tissue collected was homogenized and processed as described by Azevedo et al. [25]. The detection of TNF-α, IL-1β, and IL-10 concentrations were determined by ELISA.

Briefly, microtiter plates were coated overnight at 4°C with antibody against murine TNF-α, IL-1β, and IL-10 (2 μg/ml). After blocking the plates, the samples and standard at various dilutions were added in duplicate and incubated at 4°C for 2 h.

The plates were washed three times with a buffer. After washing the plates, biotinylated sheep polyclonal anti-TNF-α or anti-IL-1β or anti-IL-10 (diluted 1:1000 with assay buffer 1% BSA) was added to the wells.

After further incubation at room temperature for 1 h, the plates were washed and 50 μl of avidin-HRP diluted 1:5000 were added. The color reagent o-phenylenediamine.

(OPD; 50 μl) was added 15 min later and the plates were incubated in the dark at 37°C for 15–20 min. The enzyme reaction was stopped with 2 N H2SO4 and absorbance was measured at 450 nm. Values were expressed as picograms/milliliter (pg/ml).

### Gut-to-blood bacterial translocation

Since inflammatory-induced mucosa injury may cause increased gut-to-blood bacterial translocation, we measured bacterial translocation to the spleen, as this organ is involved in blood bacterial clearance. After sacrifice, an incision was made in the midline of the animal and the viscera were exposed. Then, a sample from the spleen tissue was collected to assess gut-to-blood bacterial translocation. The fragments were weighed and triturated with 1 mL of PBS. From this homogenate, 200 μL were seeded in culture MacConkey agar medium, staying at 37°C for 48 h. After incubation, the plates were photographed using an inverted microscope coupled with a CCD camera, and the images were analyzed with the Image Pro-Plus 5.0 software in order to quantify the colony forming units (CFU).

### Intestinal morphometry

Villus height and crypt depths were measured from slides stained with hematoxylin and eosin on a light microscope (BH-2, Olympus, Tokyo, Japan), n = 6, for each group, equipped with a high-resolution digital camera that was connected to a computer with an image capture program. Villus height was measured from the baseline to the villus tip. The crypt depth was measured from the baseline to the crypt bottom. A villus/crypt ratio was calculated to further address absorptive intestinal area. At least 10 clear longitudinal sections of villi and crypts were selected and counted for each sample (6 jejunal samples for each group). All morphometric measurements were done blindly by the NIH Image J 1.34 S software (National Institutes of Health, Bethesda, MD).

### Western blot

In brief, jejunal segments were harvested and immediately frozen in liquid nitrogen. Thawed specimens were pulverized in glass homogenizers, containing lysis buffer and then transferred to test tubes with protease inhibitor and centrifuged at 14000 rpm for 10 minutes. Supernatants were assayed using the bicinchoninic acid method, BCA Protein Assay Kit (Pierce, Rockford, IL) to standardize 50 μg of protein product in each well. Samples were loaded into 10% denaturing polyacryamide gels (Amersham Biosciences, UK), and gels were transferred overnight and then blotted onto nitrocellulose membranes. Membranes were blocked overnight (5% fat-free milk solution), incubated with rabbit villin (1:500) or β-actin (1:1000) antibody for 1 hour rinsed 3 times in rinsing buffer, incubated in a biotinylated secondary antibody (Horseradish Peroxidase, 1:1000), and then rinsed as described above. Each membrane was washed with cumaric acid, luminol, Tris e H_2_O_2_ and exposed to Kodak X-Omat AR film (Kodak, Rochester, NY). Western blot bands were identified and the densitometry analyzed by Image J (Media Cybernetics, CA, USA), and were expressed as villin/β-actin ratio.

### Immunohistochemistry

The immunohistochemistry targeted villin, a 92.5 kDa actin-binding protein, and one scaffold component of the intestinal microvilli in order to additionally evaluate the absorptive intestinal area. Jejunal samples were sectioned in 5 μm-thick cross-sections, placed in 10 mM citrate buffer of pH 6.0, and heated for 10 min. Sections were incubated for 15 min in 3% hydrogen peroxide (SigmaAldrich), washed with distilled water and phosphate buffered saline for 5 min each, permeabilized in 0.3% Triton (SigmaAldrich) for 15 min and in 0.1% Tween (Fisher Scientific, Pittsburg, MA) for 5 min, blocked in 10% normal goat serum in PBS for 1 h at room temperature (RT), and then incubated overnight (4°C) with rabbit primary polyclonal villin antibody (Santa Cruz) diluted in PBS-BSA. Afterwards slides were incubated in biotinylated anti-rabbit secondary antibody in PBS-BSA and incubated with Vectastain Elite ABC Reagent, according to the manufacturer’s protocol (Vector Laboratories). After washings, the section was developed with diaminobenzidine substrate ki, 3,3 = diaminobenzidine (Vector Laboratories), to give a brown to gray/black color. Slides were counterstained with methyl green, dehydrated in serial ethanol and xylene solution and permanently mounted.

### Goblet cell count

The slides used for immunohistochemistry were further used for goblet cell counting. Goblet cells were counted on a light microscope at high magnification (Olympus, CX31, Tokyo, Japan) and the data were expressed as number of cells/villus, as goblet cell villus index. The slides were viewed under a light microscope, equipped with a high definition camera, and connected to a computer with software to capture images (Q-capture, Olympus, Tokyo, Japan). At least 20 intact villi were used for goblet cell index for each rat jejunum (400X).

### Statistical analysis

Statistical analyses were performed with the aid of GraphPad Prism software, version 5.00 for Windows (San Diego, Calif., USA). Results were expressed as mean ± standard error of the mean (SEM). For the results of weight gain, serum zinc, ELISA, CBC, morphometry, western blot and goblet cell count, comparisons between groups were made using analysis of variance (ANOVA) followed by Bonferroni’s test. Diarrheal scores were analyzed by nonparametric Mann–Whitney test. Bacterial translocation data were analyzed using the Kruskal-Wallis test with Dunn's correction and Mann–Whitney test when appropriate. The value of *P* <0.05 was considered significant.

## Results

### Weight gain

**Figure 2:**
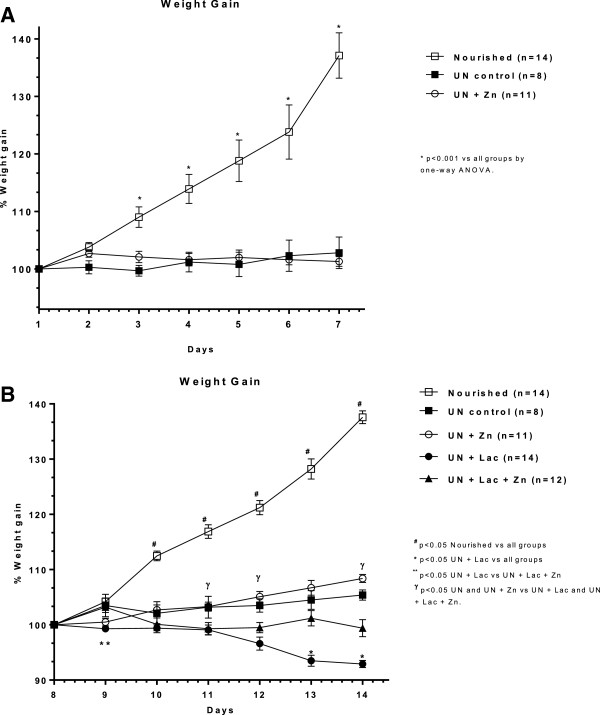
**Weight curves from experimental Wistar rats A. after challenged by the regional basic northeast diet (RBD) and B. following lactose administration.** The RBD was given *ad libitum* for 14 days and lactose (30 g/Kg, by gavage) was administered after the 8th day of the experiment. Results are expressed in mean ± SEM and were analyzed by one-way ANOVA, followed by Bonferroni’s multiple test. Groups: nourished control, undernourished control (UN), undernourished control supplemented with zinc (UN + Zn), undernourished control challenged by lactose (UN + Lac), and lactose-challenged undernourished rats supplemented with zinc (UN + Zn + Lac).

RBD caused a significant decrease in weight gain since day 3 in the undernourished control group (~9% decrement) compared with nourished controls (p < 0.001). Undernourished animals receiving RBD barely gained weight during the first seven days of the experiment. Zinc supplementation (500 mg/L) was not able to improve weight gain during the RBD challenge, compared with undernourished group without zinc supplementation (Figure 2A). Oral saturated lactose (30 g/kg by gavage, from the 8^th^ day of experiment) induced significant weight loss after the 10^th^ day (p < 0.05) in undernourished rats coinciding with the onset of osmotic diarrhea, compared with the undernourished group without lactose. After the 10^th^ day, the weight curves of undernourished rats receiving lactose and the undernourished group receiving lactose and zinc began to diverge. The undernourished groups without lactose (UN and UN + Zn) showed better gain weight than groups challenged by lactose at days 11, 12 and 14. Zinc supplementation prevented weight loss in animals challenged with lactose at the last days of the experiment (days 13 and 14) (p < 0.05), Figure 2B.

### Serum zinc levels

RBD challenge caused significant reductions in serum zinc levels compared with the nourished controls (p < 0.05). Zinc supplementation (500 mg/L) improved zinc serum levels in the rats with or without lactose challenge. The groups that received lactose with or without zinc supplementation showed higher serum zinc levels, compared with their respective controls (p < 0.01), Table 1.

**Table 1:** Zinc serum levels and jejunal morphometrics in experimental rats challenged by lactose-induced diarrhea and the regional northeast basic diet with their respective controls

	**Nourished**		**Undernourished**					
**Outcomes biochemisty**	**PBS**	**PBS**	***P***	**Zn**	***P (Zn vs PBS)***	**Lac**	**Zn + lac**	***P (Zn + Lac vs Lac)***
Zinc serum levels (mg/L) (n)	1.089 ± 0.08 (n = 11)	0.712 ± 0.10 (n = 10)	< 0.0001	1.57 ± 0.12 (n = 8)	< 0.0001	1.12 ± 0.24 (n = 12)	2.04 ± 0.50 (n = 8)	< 0.0001
Jejunal morphology*								
Villus height (μm)	826.3 ± 92.2	587.9 ± 98.3	< 0.0001	704.2 ± 113.9	0.001	686 ± 103.2	667.9 ± 78.2	0.359
Crypt length (μm)	151.5 ± 36.5	187.7 ± 66.7	< 0.0001	169.8 ± 49.0	0.007	141.6 ± 27.7	127.2 ± 20.3	< 0.0001
Villus/crypt ratio	6.03 ± 1.7	3.7 ± 1.4	< 0.0001	5.15 ± 2.82	0.0322	5.1 ± 0.8	5.4 ± 1.19	0.235

*At least 40 sections of jejunal villi and crypts stained with hematoxylin and eosin in at least four animals per group. Data are presented as mean ± SD. Comparisons were performed by Student’s unpaired *t* test. Villi and crypts were measured only when their full longitudinal axis was found.

Zn = zinc treated-rats; Lac = lactose challenged-rats.

### Diarrhea scores

RBD induced malnutrition did not cause osmotic diarrhea. Undernourished and lactose challenged rats showed diarrhea since the second challenge day indicated by the significant increase in diarrheal score (p < 0.05). From the 10th day of the experiment, animals receiving zinc supplementation showed a significant reduction in diarrhea scores, compared with the challenged untreated group, Table 2.

**Table 2:** Oral zinc supplementation reduced diarrheal scores following lactulose-induced osmotic diarrhea

**Day**	**UN N = 11**	**UN + Zn N = 12**	***P***	**UN + Lac N = 12**	**UN + Lac + Zn N = 12**	***P***
**8**	0 (0–0)	0 (0–0)	ns	0 (0–0)	0 (0–0)	ns
**9**	0 (0–0)	0 (0–0)	ns	2 (0–4)	1 (0–4)	ns
**10**	0 (0–0)	0 (0–0)	ns	1 (0–3)	0 (0–1)*	<0.05
**11**	0 (0–0)	0 (0–0)	ns	2 (1–4)	0.5 (0–4)*	<0.05
**12**	0 (0–0)	0 (0–0)	ns	2 (1–4)	0.5 (0–4)*	<0.05
**13**	0 (0–0)	0 (0–0)	ns	2 (0–4)	0 (0–4)*	<0.05
**14**	0 (0–0)	0 (0–0)	ns	2 (1–4)	1 (0–2)*	<0.05

Normal stools: normal texture, consistency, and dryness.

Diarrhea scores: + = wet stools; ++ = pasty stools; +++ = semiliquid stools; ++++ = watery diarrhea.

Data are reported as median score. The score total range is shown in parenthesis. Data were analyzed by Kruskal-Wallis and Dunns post-test.

Groups: UN = undernourished controls; UN + Zn = undernourished rats treated with oral zinc acetate (500 mg/L); UN + Lac = undernourished rats challenged by saturated lactose (30 g/Kg); UN + Lac + Zn = Zinc-treated undernourished rats challenged by saturated lactose (30 g/Kg).

### TNF-α, IL-1β and IL-10 tissue levels

**Figure 3:**
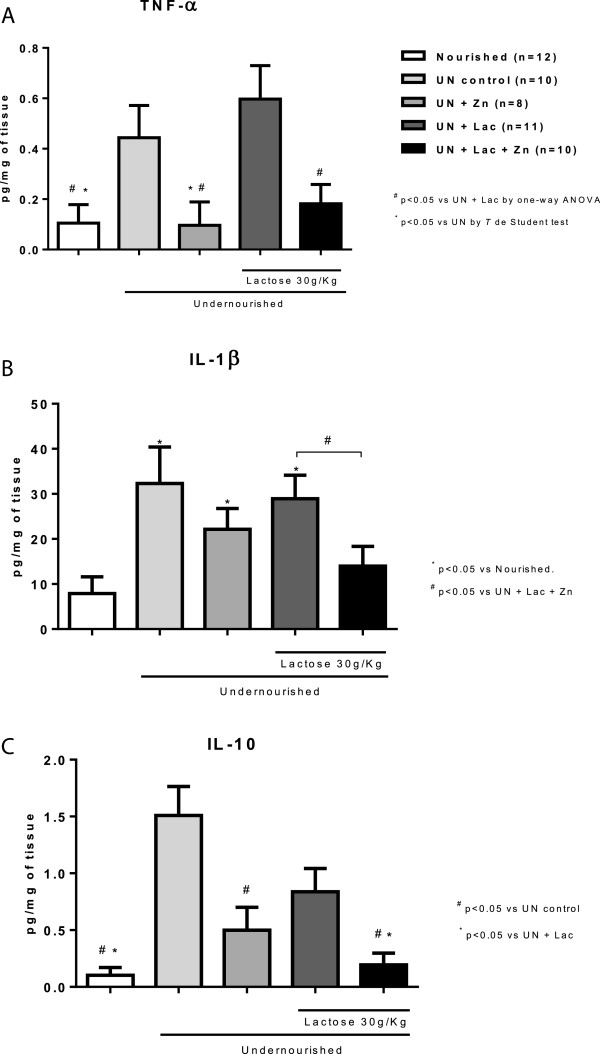
**Jejunal cytokine assays for (A) tumor necrosis factor-alpha (TNF-α), (B) interleukin-1 beta (IL-1 β), and (C) interleukin-10 (IL-10) from experimental Wistar rats challenged by the regional basic northeast diet (RBD) and following lactose administration.** The RBD was given *ad libitum* for 14 days and lactose (30 g/Kg, by gavage) was administered after the 8th day of the experiment. Cytokines were measured from jejunum homogenates by ELISA. Values represent mean ± SEM and were analyzed by one-way ANOVA. At least four animals were used per group. At least N = 8 for all groups. Groups: nourished control, undernourished control (UN), undernourished control supplemented with zinc (UN + Zn), undernourished control challenged by lactose (UN + Lac), and lactose-challenged undernourished rats supplemented with zinc (UN + Zn + Lac).

Tissue levels of TNF-α (p < 0.05), IL-1β (p < 0.05) and IL-10 (p < 0.001) were increased in the jejunum from undernourished rats compared with nourished controls. Zinc supplementation (500 mg/L) reduced tissue levels of TNF-α (p < 0.05) and IL-10 (p < 0.01) compared with the undernourished group without zinc supplementation, but did not reduce jejunal IL-1β levels. Lactose challenge (30 g/Kg) significantly increased TNF-α (p < 0.05), IL-1β (p < 0.05), and IL-10 (p < 0.01) levels, compared with nourished controls. Zinc supplementation to the lactose-challenged group reduced tissue TNF-α, IL-1β, and IL-10 (p < 0.05), compared with the lactose-challenged untreated undernourished controls, Figure 3.

### Intestinal bacterial translocation

**Figure 4:**
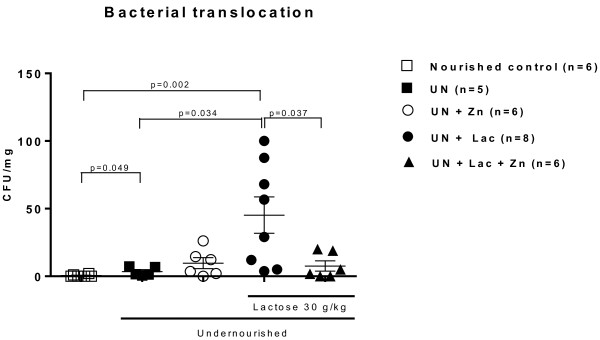
**Bacterial translocation experiments from Wistar rats challenged by the regional basic northeast diet (RBD) and following lactose administration.** The RBD was given *ad libitum* for 14 days and lactose (30 g/Kg, by gavage) was administered after the 8th day of the experiment. Values are expressed in aerobic colony-forming units (CFU) per milligram of the spleen tissue. Statistical analyses were done using Mann-Whitney or Kruskal-Wallis followed by Dunns’ correction. Groups: nourished control, undernourished control (UN), undernourished control supplemented with zinc (UN + Zn), undernourished control challenged by lactose (UN + Lac), and lactose-challenged undernourished rats supplemented with zinc (UN + Zn + Lac).

Undernourishment caused significant intestinal bacterial translocation (BT) to the spleen (p < 0.05). Zinc treatment was unable to improve bacterial translocation to the spleen in RBD-challenged rats. Lactose challenge caused a significant increase in BT to the spleen compared to undernourished (p = 0.03) and nourished controls (p = 0.002). Zinc treatment significantly decreased the incidence of BT in groups challenged by lactose (p < 0.05) (Figure 4).

### Intestinal morphometry

Villus height was significantly reduced in all undernourished groups compared with the nourished controls (p < 0.0001). On the other hand, crypt depth was significantly increased in undernourished controls compared with the nourished group (p < 0.0001). Villus/crypt ratio was lower in the undernourished control compared with nourished counterparts.

Zinc supplementation increased villus height (p < 0.0001), crypt depth (p < 0.01), and villus/crypt ratio in undernourished rats, compared to untreated controls, even greater than nourished controls (p < 0.001).

The lactose administration increased villus height (p < 0.01), reduced crypt depth (p < 0.0001), and improved villus/crypt ratio in undernourished mice. Zinc treatment increased villus/crypt ratio and reduced crypt depth in lactose-challenged undernourished rats (Table 1).

### Intestinal villin assessment

**Figure 5:**
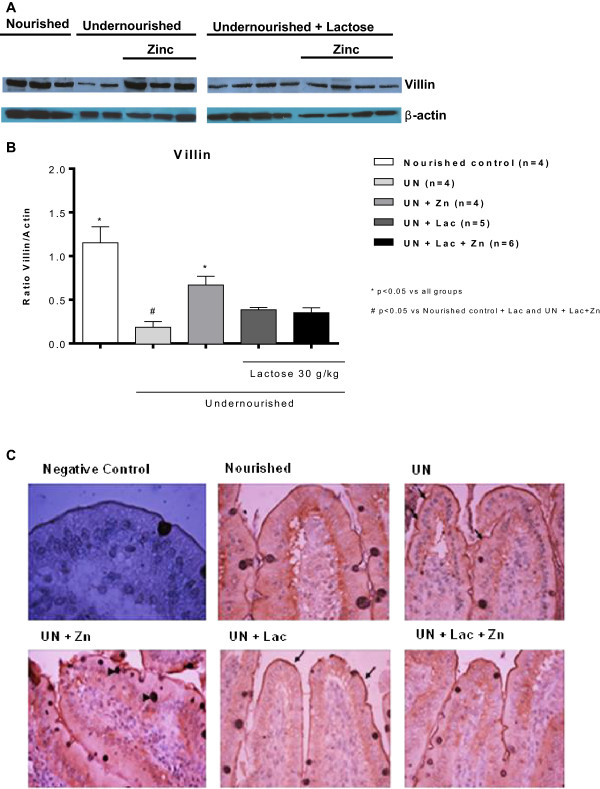
**Villin jejunal expression. A**. Representative villin immunoblots from snap-frozen jejunal tissue and **B**. villin:β-actin ratio band densitometry from experimental Wistar rats on the regional basic northeast diet (RBD) and following lactose administration. **C**. Representative jejunal villin immuhistochemistry from experimental Wistar rats. Note increase in microvilli thickness in the undernourished group (arrows). Arrow head: goblet cells. X400. The RBD was given *ad libitum* for 14 days and lactose (30 g/Kg, by gavage) was administered after the 8th day of the experiment. Values were analyzed by one-way ANOVA and *T* Student.

Jejunal villin expression was significantly reduced (p < 0.0001) with RBD-induced malnutrition as identified by western blot (Figure 5A, B), but with increased brush border thickness, compared with the nourished group (Figure 5).

Zinc supplementation increased villin expression (p < 0.05) and the intensity of brush border villin imunostaining.

Lactose administration increased the intestinal villin expression, compared with unchallenged undernourished rats (p < 0.05), however, with a decrease in both the thickness and intensity of villin immunostaining, similarly to the nourished group.Zinc supplementation did not change jejunal villin expression in lactose-challenged rats, but clearly increased brush border thickness and immunostaining (Figure 5).

### Villus goblet cell count

**Figure 6:**
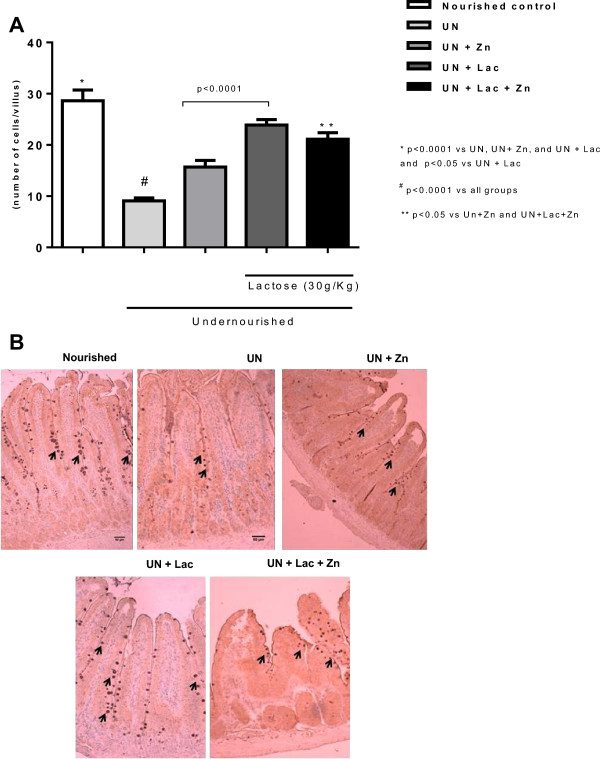
**Jejunal histology of Wistar rats challenged by the regional basic northeast diet (RBD) and following the lactose administration. A**. Goblet cell villus index and **B**. Representative jejunal histological sections from the experimental groups. Arrows indicate goblet cells. Methyl-green, x400. The RBD was given *ad libitum* for 14 days and lactose (30 g/Kg, by gavage) was administered after the 8th day of the experiment.

Goblet cell count was reduced in the undernourished control group compared to the nourished controls (p < 0.0001). Zinc supplementation significantly increased villus goblet cell numbers (p < 0.01), to the level of the nourished group.Lactose administration increased goblet cell count compared to either zinc treated or untreated controls (p < 0.001), although being lower than the nourished group (p < 0.05) (Figure 6).

## Discussion

In our model, a multideficient rodent diet (regional basic diet, RBD) promotes malnutrition and zinc deficiency similarly to what is seen in undernourished children afflicted with heavy burden of diarrhea in northeastern Brazil. In a study conducted in rural South Africa, children who were fed with a high-carbohydrate diet (70% of total energy) and deprived in most micronutrients (including zinc), showed delayed linear growth [26].

Although the RBD, enriched in carbohydrate and with low protein, has been used in several studies to induce malnutrition [27,28], our study is the first to evaluate a dual hit of RBD-induced malnutrition and osmotic diarrhea, along with the role of zinc as a gut-trophic nutrient to improve intestinal inflammation, intestinal bacterial translocation, and intestinal morphology.

We have shown that the RBD caused an intestinal inflammatory state, characterized by increased pro-inflammatory intestinal cytokine levels (IL-1β and TNF-α), that is ameliorated by zinc supplementation. A recent study by de Oliveira Assis and colleagues reports that administration of the RBD during gestation and lactation to dams was associated with a reduced systemic inflammatory response in the offspring [29], suggesting that the timing and degree of intestinal maturation, change in diets, and intestinal milieu play a role in modulating intestinal inflammatory responses.

Our findings also provide evidence of the importance of zinc supplementation during early post-natal growth. In agreement with our findings, a study with weanling pigs found that supplementation with zinc oxide promoted a greater daily weight gain (p < 0.001), compared with the untreated group [30]. In humans, a recent meta-analysis on preventive zinc supplementation in children highlighted significant improvements in weight gain and linear growth over time [31].

In this study, lactose-induced diarrhea in undernourished rats amplified the RBD-negative effects on weight gain, probably due to increased fluid loss hindered by osmotic diarrhea. In earlier studies, oral lactose (30 g/kg) produced weight loss and osmotic diarrhea to rats, with intestinal barrier leakage, observed by increased jejunal permeability to horseradish peroxidase (HRP) [22].

We treated rats with zinc concomitantly with RBD-induced malnutrition; hence, this might explain a lack of zinc effect in improving weight gain. However, our study reinforces the importance of zinc supplementation on weight gain in the presence of diarrhea, suggesting the role of zinc in intestinal fluid and electrolyte balance and intestinal barrier integrity as mechanism to prevent weight loss. In a meta-analysis in pooled 33 randomized trials (27 in developing countries) conducted between 1976 and 2000, zinc supplementation results in a positive response in linear growth and weight gain in children. This benefit was more significant in undernourished children, emphasizing that the positive growth response is more readily apparent in previously stunted children [31].

The possible mechanisms by which zinc supplementation improves diarrhea include increased absorption of water and electrolytes by the gut [32], intestinal epithelial regeneration or restoration of function [33,34], increased levels of enterocyte brush border enzymes [35,36] and the enhancement of the mucosal immune response, including cellular immunity and higher levels of secretory antibodies [37].

One unexpected result was increased serum zinc levels in lactose-challenged undernourished rats, since serum zinc was higher in undernourished groups with lactose when compared with controls (UN + Lac > UN, p < 0.05 and UN + Lac + Zn > UN + Zn, p < 0.05). Lactose effects in mineral absorption were examined by Greger and colleagues [38], using weaned male rats, fed with different diets containing lactose. Groups that received high lactose diets showed greater bone zinc absorption, as measured by atomic absorption spectroscopy of the tibia. The authors suggest that lactose increased zinc intestinal absorption; however serum zinc levels were not significantly altered [38]. In a study assessing zinc transport through the brush border membrane vesicles of intestinal biopsies from neonatal piglets, lactose (50 mM, 18 g/L) significantly stimulates zinc transport with a 150% increase in zinc uptake by the group that received lactose compared with control group (p < 0.05) [39].

Lactose intestinal absorption first requires the action of lactase (that breaks the bond between glucose and galactose) presented in the brush border of the small intestine [40]. However, after weaning, when lactose is no longer an essential component in the diet of mammals, there is a genetically programmed and irreversible reduction in lactase activity [41]. Thus, saturated oral lactose administration can cause a hyperosmolar luminal gradient, changing the jejunal epithelial permeability and resulting in osmotic diarrhea.

In a study enrolled with eighty children aged one to five years with moderate and severe malnutrition showed increased TNF-α and IL-10 serum levels [42]. In contrast, in another study, the ability to produce the anti-inflammatory cytokine IL-10 was found decreased in undernourished children [43]. In Monk and Woodward (2009), post-weaning mice that received a low-protein diet (0.6%) showed high IL-10 serum levels compared to controls. Despite the inadequate supply of energy, the production of systemic IL-10 was maintained in the undernourished groups, and, in fact, appeared higher in the group with marasmus (p = 0.05) during the progression of weight loss [44]. In contrast, male mice with two months of life, challenged with low-protein diet (4%, compared with 20% of the standard diet) and LPS showed reduced synthesis of TNF-α and IL-1β [45]. In our study, the high percentage of carbohydrates in RBD may have contributed to the high level of TNF-α. According to Ferreira et al. [46], Swiss mice, with 7–8 weeks that received high calorie diet (64% carbohydrates and 19% protein) showed hyperglycemia, with lower glucose tolerance and increased level of TNF- α, both in plasma and in the liver [46].

Zinc plays a role in acute-phase response and immune response [47]. It is also known to accelerate IL-1β secretion, which inhibits the development of BT in mice subjected to shock [48]. IL-1β is associated with activation of guanylate cyclase and uroguanylin that activate the cystic fibrosis transmembrane conductance regulator (CFTR), an important chloride secretagogue present in intestinal glands, and therefore regulate water crossing to the intestinal lumen. The anti-inflammatory and anti-IL-1β effects of zinc in our model could have reduced CFTR activation on the intestinal mucosa and therefore ameliorate the lactose-induced osmotic diarrhea.

Alternatively, the change in intestinal permeability may have been the result of a process mediated by the intestinal microbiome. The bacterial population in the intestinal lumen may have been changed with excess of free carbohydrates and lactose as the carbohydrates are presented in greater quantity in RBD. These carbohydrates may have been used as substrates for bacterial overgrowth and generation of its products, as discussed by Bui and colleagues [48].

Zinc supplementation may have had a beneficial effect on the intestinal morphology by increasing the expression of IGF-1 in the mucosa of the small intestine, resulting in increased mucosal growth, brush border enzymatic activity, and nutrient uptake [30,49]. A recent study has shown the benefit of oral zinc supplementation in improving IGF-1 plasma levels and growth in children [50]. Another possible explanation is the role of zinc in the synthesis of RNA and DNA. This increase can be explained by increased cell proliferation and protein synthesis promoted by zinc supplementation [51].

Villin is a structural component of microvilli forming the brush border of the small intestine. The villin plays a key role in maintaining the brush border organization by binding to F-actin in a network of filaments. Studies with knock-out animals for villin showed that although this protein is not needed to form bundles with F-actin, it is a major factor in the reorganization of the actin cytoskeleton in stress conditions [52]. Our findings have shown higher expression of villin with consequent reductions of villus height, likely to compensate for absorption loss. Zinc supplementation increased villus height and villin expression. Zinc has previously been shown to enhance intestinal absorptive surface in two ways: by improving villus height (shown by the increase of the villus/crypt ratio) and by improving brush border villin expression. It has been shown that zinc supplementation increases the number of intestinal brush border enzymes [53].

In our early studies with compound effects of undernutrition and *C. parvum* infection in neonatal mice, undernourished mice showed villus blunting, crypt atrophy, and goblet cell reductions. On the other hand, undernourished and *C. parvum* infected mice showed crypt depth hyperplasia and intestinal inflammation, and also goblet cell reductions [54]. The increase in goblet cells seen with zinc supplementation may be indicative of improved innate responses to luminal bacterial translocation. Zinc is a powerful regulator of this gene expression in the small intestine [51,55]. Furthermore, there is a continuous, dynamic interaction between the intestinal bacterial population and the mucosa layer. Luminal bacteria can stimulate the expression of genes regulating mucin secretion, causing an increase in goblet cell proliferation [55].

Although our findings suggest the benefit of zinc supplementation to lactose-induced osmotic diarrhea in rats, we acknowledge that this model may not necessarily induce the same level, mechanisms, and characteristics of the inflammatory condition seen with different pathogenic-driven-diarrhea in children. On the other hand, transient lactase deficiency secondary to brush border damage from gastrointestinal infections is a well-recognized clinical entity that prolongs diarrheal symptoms in the aftermath of acute infections [56]. Hence, amelioration of osmotic diarrhea may account, in part, for zinc's benefits in the setting of secretory and inflammatory diarrhea that damage intestinal villi. It is noteworthy that antimicrobial multi-resistant *E. coli* was documented with zinc supplementation [57].

The clinical-based diarrheal scoring method used in our study may have not account for the microbiome effects on stool characteristics. Further studies are warranted to better evaluate the effects of zinc on the intestinal microbiome in rats challenged by diarrhea and undernutrition.

In addition, our data were obtained from male rats and therefore we could not appreciate sex-specific benefits of zinc supplementation. A recent study from our group has shown that zinc supplementation combined with glutamine and vitamin A benefit shantytown girls at risk for enteric infections in the verbal learning testing [58]. Another study elsewhere has shown that girls suffering from diarrhea recovered faster than boys after zinc supplementation [59].

## Conclusion

In summary, our data support the importance of diet in regulating the intestinal milieu in response to inflammatory conditions in weanling rats subjected to undernutriton and osmotic diarrhea. This reinforces the anti-inflammatory role of zinc in promoting homeostasis of the intestinal mucosa. That has potential implications for ameliorating environmental-driven enteropathy. Therefore, altogether our findings support the use of zinc supplementation to break the vicious cycle of undernutrition and diarrhea in children and its deleterious lasting effects.

## Competing interest

All authors declare no competing of interest.

## Authors’ contributions

CCAQ, RLG, AAML, SRM, and RBO contributed with the study design, data analyses, and manuscript preparation. CCAQ, ILF, and PBF conducted animal handling and molecular biology analyses. SGCF worked in the diet manipulation and production. CCAQ and CBMC were responsible for the bacterial translocation experiments. CECM, RAR, and KSA conducted the serum zinc measurements and ELISA studies. All authors read and approved the final manuscript.

## Pre-publication history

The pre-publication history for this paper can be accessed here:

http://www.biomedcentral.com/1471-230X/14/136/prepub
